# Thrombomodulin Promotes Corneal Epithelial Wound Healing

**DOI:** 10.1371/journal.pone.0122491

**Published:** 2015-03-27

**Authors:** Yi-Hsun Huang, Ching-Chang I, Cheng-Hsiang Kuo, Yun-Yan Hsu, Fang-Tzu Lee, Guey-Yueh Shi, Sung-Huei Tseng, Hua-Lin Wu

**Affiliations:** 1 Institute of Clinical Medicine, National Cheng Kung University Medical College, Tainan, Taiwan; 2 Department of Ophthalmology, National Cheng Kung University Hospital, College of Medicine, National Cheng Kung University, Tainan, Taiwan; 3 Cardiovascular Research Center, National Cheng Kung University Medical College, Tainan, Taiwan; 4 Department of Biochemistry and Molecular Biology, National Cheng Kung University Medical College, Tainan, Taiwan; Cedars-Sinai Medical Center; UCLA School of Medicine, UNITED STATES

## Abstract

**Purpose:**

To determine the role of thrombomodulin (TM) in corneal epithelial wound healing, and to investigate whether recombinant TM epidermal growth factor-like domain plus serine/threonine-rich domain (rTMD23) has therapeutic potential in corneal epithelial wound healing.

**Methods:**

TM localization and expression in the murine cornea were examined by immunofluorescence staining. TM expression after injury was also studied. The effect of rTMD23 on corneal wound healing was evaluated by in vitro and in vivo assays.

**Results:**

TM was expressed in the cornea in normal adult mice. TM expression increased in the early phase of wound healing and decreased after wound recovery. In the *in vitro* study, platelet-derived growth factor-BB (PDGF-BB) induced TM expression in murine corneal epithelial cells by mediating E26 transformation-specific sequence-1 (Ets-1) via the mammalian target of rapamycin (mTOR) signaling pathway. The administration of rTMD23 increased the rate of corneal epithelial wound healing.

**Conclusions:**

TM expression in corneal epithelium was modulated during the corneal wound healing process, and may be regulated by PDGF-BB. In addition, rTMD23 has therapeutic potential in corneal injury.

## Introduction

The cornea is the transparent tissue of the eye that consists primarily of three cellular and two acellular layers. The outermost layer is the corneal epithelium, followed by Bowman’s membrane, stroma, Descemet’s membrane, and endothelium. After corneal injury, epithelial cells from the basal epithelial layer and from the limbus proliferate and migrate to cover the wound bed before differentiating into new multilayered epithelium [1].

In general, successful wound healing involves various processes including cell migration, proliferation, cell-matrix adhesion, and tissue remodeling, which are often driven by growth factors and other factors released coordinately into the injured area by epithelial cells [2, 3]. In the cornea, prominent factors include epidermal growth factor (EGF), transforming growth factor-β (TGF-β), platelet-derived growth factor (PDGF), and interleukin-1 [4]. Other factors such as adenosine triphosphate (ATP) and lipid mediator lysophosphatidic acid are released from the injured cells to enhance epithelial migration and wound healing in the cornea [5]. Inflammation is another important factor in the wound healing process. It is an early response to corneal damage and an integral part of wound healing; however, sustained corneal inflammation may lead to opacity, ulceration, stromal melting, and tissue destruction [6, 7]. While the basic elements of the healing process are known, the detailed mechanism and associated factors thereof remain under investigation.

Thrombomodulin (TM) is a transmembrane glycoprotein originally identified in vascular endothelium [8]. It consists of five domains, including a highly charged N-terminal lectin-like domain (D1), a domain with six EGF-like structures (D2), a serine and threonine-rich domain (D3), a transmembrane domain (D4), and a cytoplasmic domain (D5) [9, 10]. TM is a multifunctional protein that performs distinct functions in different cell types. In the endothelium, it inhibits the procoagulant effects of thrombin, and significantly enhances the thrombin-dependent activation of anticoagulant protein C [11]. In keratinocytes, TM expression is regulated during differentiation [12]. In cutaneous wound healing, the release of soluble TM from keratinocytes may promote wound healing [13]. Furthermore, the recombinant TM EGF-like domain plus a serine/threonine-rich domain, rTMD23, promoted cutaneous healing in a murine model of full-thickness wound healing [14].

TM localization in the human eye was reported by Ikeda et al. [15]. In that study, TM was detected in corneal epithelial cells, limbus, corneal endothelial cells, lens epithelial cells, trabecular meshwork, nonpigmented ciliary epithelial cells, and Schlemm’s canal. The authors suggested that TM may regulate cell proliferation or differentiation [15]. In addition, the role of TM has been investigated in inflammatory eye diseases such as endophthalmitis and herpetic keratitis [16]. The results showed that TM was expressed in the corneal epithelium and stromal cells in inflammatory eye diseases. TM may be regulated by PDGF-BB in corneal stromal cells, and TM may regulate corneal inflammation along with other cytokines [16]. While evidence suggests that TM may regulate cell proliferation and corneal inflammation [15, 16], its role in corneal wound healing remains unclear. Thus, in this study we investigated TM expression patterns in the murine cornea, and the role of TM in corneal epithelial wound healing. Furthermore, we examined the therapeutic potential of recombinant TM proteins in corneal epithelial damage.

## Materials and Methods

### Ethics statement

Animal experiments were performed in accordance with the ARVO Statement for the Use of Animals in Ophthalmic and Vision Research, and the protocol was approved by the Institutional Animal Care and Use Committee of National Cheng Kung University, and conformed to the Guide for the Care and Use of Laboratory Animals published by the National Institutes of Health (NIH Publication #85–23, revised 1996).

### Recombinant TM protein expression and purification

rTMD23 expression and purification in the *Pichia pastoris* expression system was conducted as previously described [17, 18]. Recombinant proteins were purified using nickel-chelating Sepharose columns (Amersham Pharmacia Biotech, Uppsala, Sweden) and examined by sodium dodecyl sulfate-polyacrylamide gel electrophoresis (SDS-PAGE).

### Western blot analysis

Approximately 10 μg of total protein was separated via 10% SDS-PAGE, and transferred onto a polyvinylidene difluoride membrane. The membranes were blocked with 5% nonfat milk powder for 1 h at room temperature. After being probed with specific antibodies (all from Santa Cruz Biotechnology) against TM (sc-7097), cytokeratin 14 (sc-53253), cytokeratin 12 (sc-17101), E26 transformation-specific sequence-1 (Ets-1, sc-350), cytokeratin 5 (sc-66856), and glyceraldehyde 3-phosphate dehydrogenase (GAPDH, sc-32233) at 4°C overnight, the membranes were incubated with peroxidase-conjugated specific secondary antibodies (Calbiochem EMD Biosciences, Inc., La Jolla, CA) for 1 h at room temperature. The signal was detected using an enhanced chemiluminescence reagent (Millipore, Billerica, MA) and a Fujifilm LAS-3000 imager (Fujifilm Life Science, Stamford, CT).

### Reverse transcription-polymerase chain reaction (RT-PCR)

Total RNAs were extracted from the scraped mouse corneal epithelium using the High Pure RNA Isolation Kit (Roche Diagnostics Ltd., Mannheim, Germany). PCR primers for TM and β-actin were as follows: TM-forward, 5′-CTTGTGCAATAGGAGCACGA-3′, TM-reverse, 5′-GACACAAAAATGCTCGCAGA-3′; β-actin-forward, 5′-TGTTACCAACTGGGACGACA-3′, and β-actin-reverse, 5′-GGGGTGTTGAAGGTCTCAAA-3′.

### Immunohistochemistry

Tissue samples were fixed in 3.7% formaldehyde, embedded in paraffin, and cut into 5-μm sections. For histological analysis, sections were stained with hematoxylin and eosin (H&E) or treated with rabbit anti-mouse TM antibody purified by affinity chromatography in our laboratory. Alexa Fluor 488-conjugated anti-rabbit IgG antibody (Molecular Probes, Eugene, OR) was used as a secondary antibody for immunofluorescence staining.

### Cell cultures

Primary murine corneal epithelial cells (MCECs) were isolated and cultured using methods described by Kobayashi et al. [19]. In brief, eyes removed from C57BL/6 mice were incubated overnight (18 h at 4°C) in Dulbecco’s Modified Eagle’s medium (DMEM)/F12 (1:1 mixture, GIBCO BRL, Grand Island, NY) containing 15 mg/mL Dispase II (Sigma, St. Louis, MO), 100 mM sorbitol (Sigma), and an antibiotic-antimycotic solution (Sigma). The corneal epithelium was removed as a sheet and dissociated into single cells in Accutase (Millipore, Billerica, MA). Dissociated cells were then seeded into type I collagen-coated 12-well plates and cultured in CnT-50 (CELLnTEC, Bern, Switzerland), a serum-free, low bovine pituitary extract (BPE) medium. After reaching subconfluence, cells were subcultured, and third-passage cells were used for the experiments. Human corneal epithelial cells (HCECs) were obtained from Invitrogen-GIBCO. HCECs were cultured in Keratinocyte Serum Free Medium (SFM) (Invitrogen-GIBCO) supplemented with BPE 50 μg/mL, recombinant human EGF 5 ng/mL, and antibiotic-antimycotic 1x (all from Invitrogen-GIBCO). The cells were maintained under standard cell culture conditions at 37°C with humidified air with 5% CO_2_.

### PDGF-BB and TM expression in MCECs and HCECs

To determine the relationship between PDGF-BB and TM, MCECs and HCECs were seeded onto 12-well plates. Confluent cells were starved for 24 h in SFM and then stimulated with 1.25 ng/mL, 5 ng/mL, and 20 ng/mL PDGF-BB (R&D systems). After 6 h, cells were rapidly washed twice with ice-cold PBS and lysed with 200 μL of 0.1% Triton X-100 lysis buffer (Cell Signaling Technology) containing protease inhibitor (s8830; Sigma). Total lysates were cleared by centrifugation at 13000 rpm for 15 min, and protein concentration was determined using the BCA method (Thermo-Scientific). Aliquots of 15 μg of protein/lane for each sample were analyzed by western blot with antibodies against TM (sc-13164 or sc-7097), Ets-1, and GAPDH. In our previous study, we found that PDGF-BB stimulates functional TM expression by mediating Ets-1 via the mammalian target of rapamycin (mTOR) signaling pathway [20]. To determine whether the same signaling pathway mediates PDGF-BB-induced TM expression, we investigated the involvement of mTOR in TM induction by pretreating starved MCECs with rapamycin (5 ng/mL; Sigma) for 60 min. For immunofluorescence staining, MCECs were seeded onto type I collagen-coated glass coverslips in CnT-50 medium and grown to confluence for 24 h. After growth factor starvation for 24 h in CnT-20 medium containing 5 μg/mL insulin (CnT-20-I, CELLnTEC), the medium was replaced with medium containing PDGF-BB. The cells were fixed in 3.7% paraformaldehyde (Merck KGaA, Darmstadt, Germany) after 6 h of incubation, permeabilized via 0.1% Triton X-100 (Merck KGaA) in PBS for 4 min, then washed three times in PBS. Cells were blocked with 5% goat serum (Sigma) for 1 h and incubated with rabbit anti-TM antibody overnight at 4°C. The Alexa Fluor 488-conjugated anti-rabbit IgG antibody (Molecular Probes) was used as a secondary antibody. Glass coverslips were washed three times with PBS, mounted, and examined using a Leica fluorescence microscope (Leica Microsystems, Wetzlar, Germany).

### In vitro wound healing assay

Confluent MCECs in 12-well plates were starved overnight in medium containing 5 μg/mL insulin (CnT-20-I). The surface of the plate was scraped with a 200-μL pipette tip to generate a cell-free zone. Free cells were then removed with two washes with PBS, and cells were incubated in the CnT-20-I medium containing different concentrations of rTMD23 (0, 20, and 200 ng/mL). Cells were imaged using a time-lapse video microscopy system (Olympus, Tokyo, Japan) immediately after wounding. The area of wound closure was quantitatively determined using ImageJ software (National Institutes of Health, Bethesda, MD). The percentage of wound closure at the indicated time-points was calculated by 100% minus the percentage of the denuded area to the initial scraped area.

### In vivo wound healing assay

C57BL/6 mice aged 7–8 weeks were used in this study. Mice were anesthetized with intraperitoneal injections of a combination of ketamine (10 mg/kg; Sigma) and xylazine (1 mg/kg; Sigma) prior to the procedure, and a drop of proparacaine hydrochloride 0.5% (Alcon) was applied to the cornea to deliver local anesthesia before injury. Epithelial abrasion was achieved using a 2-mm disposable biopsy punch, and the corneal epithelium inside the area was gently removed by a rust ring remover. Eight mice per group were treated and the experiments were repeated three times. Each mouse was topically treated with 5 μL (2 mg/mL) rTMD23 or 5 μL PBS at 3, 6, 9, 12, and 15 h after wounding. After 18 h, the wound area was stained with fluorescein. Wound areas were imaged with a Leica MZ7.5 microscope and quantified using ImageJ software. The percentage of wound closure was calculated by 100% minus the percentage of the denuded area to the initial scraped area. For terminal experiments, mice were euthanized by CO_2_ inhalation.

### Statistical analysis

In vitro experiments were analyzed using one-way ANOVA followed by Bonferroni’s comparison test. In vivo wound healing studies were analyzed using an unpaired t-test. Probability (*p)* values < 0.05 were considered statistically significant. Statistical analyses were performed using Prism 5.0 software.

## Results

### TM localization and expression in the murine cornea

**Fig 1:**
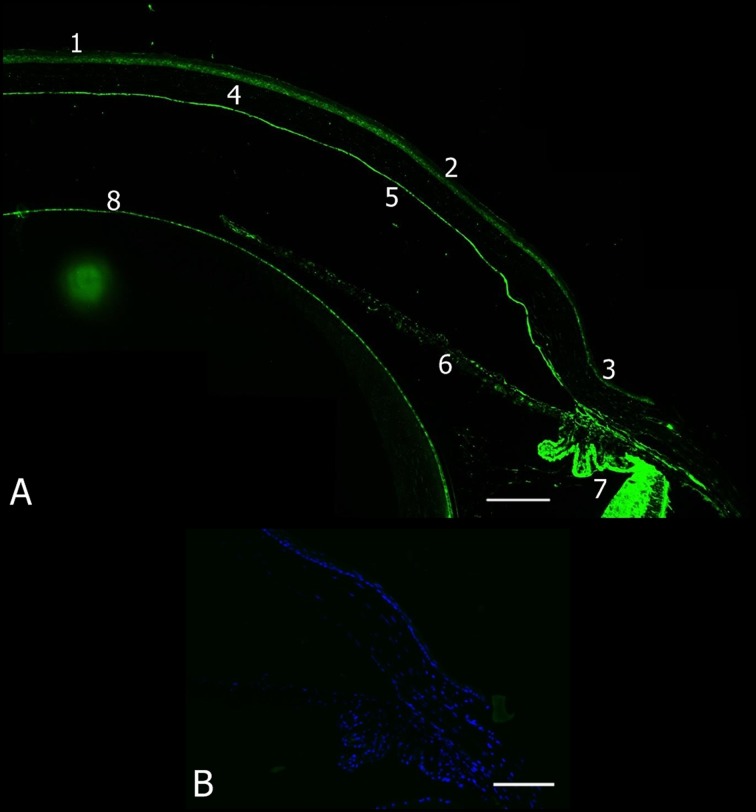
TM localization in the anterior segment of the murine eye. (A) The anterior segment of an eye from a C57BL/6 mouse was stained for TM with an anti-TM antibody (green). 1) central corneal epithelium, 2) peripheral corneal epithelium, 3) limbus, 4) corneal stroma, 5) corneal endothelium, 6) iris, 7) ciliary body, and 8) lens epithelial cell. (B) Secondary antibody only as negative control. Magnification: 200×. Bar of (A) = 250 μm and (B) = 100 μm.

**Fig 2:**
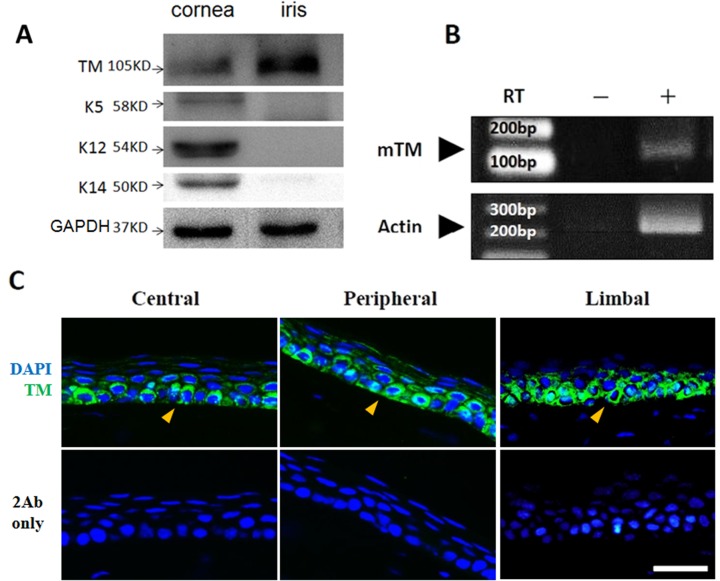
TM expression in murine corneal epithelium. *(A)* TM expression in corneal epithelium and iris were analyzed by western blot (n = 4). Tissue lysates were subjected to electrophoresis using a 10% SDS-PAGE under reducing conditions. The mouse corneal epithelium and iris both expressed TM molecules with a 105-kDa molecular mass. Epithelial cell markers such as basal cell marker cytokeratin 14 (K14), corneal differentiation marker cytokeratin 12 (K12), and epithelial cell marker cytokeratin 5 (K5) were detected with specific antibodies. *(B)* RT-PCR analysis of TM mRNA in the mouse corneal epithelium. The TM gene is intronless, and therefore, the left lane without reverse transcriptase was used as a negative control. mTM, mouse TM. *(C)* Immunofluorescence staining of TM (green) in murine corneal epithelium. The basal cells exhibited strong positive staining in the central, peripheral, and limbal corneal epithelium. Secondary antibody only (2Ab only) acted as a negative control. Nuclei (blue) were visualized by DAPI nuclear staining. Arrowheads: basal cells. Magnification: 400×. Bar = 25 μm.

To investigate TM distribution in the murine cornea, the anterior segment of eyes from C57BL/6 mice aged 7–8 weeks was stained with an anti-TM antibody. Positive immunofluorescence staining for TM was evident in the corneal epithelium, limbus, endothelium, iris, ciliary body, and lens epithelial cells, but not in the corneal stroma (Fig. 1). The scraped corneal epithelium and iris were harvested by Dispase II, and TM expression was examined by western blot and RT-PCR, which revealed that TM was expressed in the corneal epithelial cells (*n* = 4, Fig. 2A and 2B). TM localization and expression in mouse corneal epithelium was further examined by immunofluorescence using high-magnification imaging. High TM expression was apparent in the basal corneal epithelium in the central, peripheral, and limbus regions, whereas TM expression was low in wing and superficial cells (Fig. 2C). These results indicated that TM was differentially expressed in corneal epithelium.

### Increased TM expression after corneal injury

**Fig 3:**
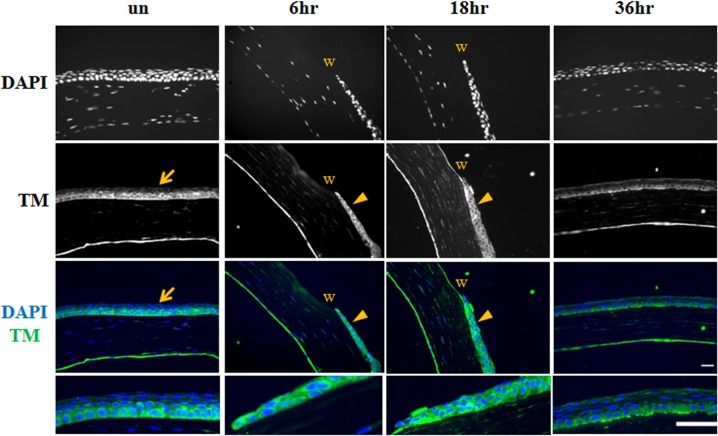
Increased TM expression in corneal epithelium injury. TM expression (green) at 6, 18, and 36 h after wounding was examined by immunofluorescence staining. Unwound (un) cornea was used as control. Arrows: TM expression in the unwounded cornea was relatively weaker compared with that in the wounded cornea. Nuclei were visualized by DAPI (blue) nuclear staining. Arrowheads: corneal epithelium; w: wounded area. Magnification: 400×. Bar = 25 μm.

To study TM expression during corneal epithelial wound healing in mice, a corneal debridement model was established by creating a central corneal wound using a biopsy punch. Wound healing was monitored by fluorescein staining, which showed that the wound area was almost completely healed 24 h after debridement (data not shown). Immunofluorescence staining showed that TM expression had increased in the entire corneal epithelium at 6 and 18 h after wounding, and decreased after wound closure (36 h, Fig. 3).

### PDGF-BB stimulated TM expression in MCECs

**Fig 4:**
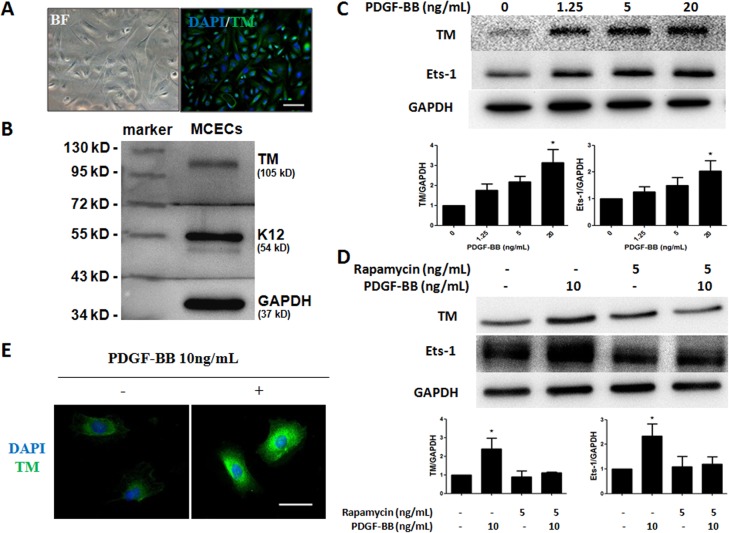
PDGF-BB stimulates TM expression in MCECs and HCECs. (A) TM expression was investigated by immunofluorescence staining. Primary MCECs were cultured in CnT-50 medium. The cells were stained by DAPI (blue) and TM (green). BF, bright field. (B) Western blot analysis of TM. MCECs lysates were analyzed by Western blot with antibodies against TM and K12. (C) HCECs were treated with various concentrations of PDGF-BB for 6 h. Expression levels of TM and Ets-1 were analyzed with Western blot and normalized by GAPDH. Western blots from three independent experiments were analyzed by densitometry. The indicated fold changes represent the density relative to control (0), * p < 0.05 compared with 0 ng/mL PDGF-BB. (D) Quiescent MCECs were treated with rapamycin (0 or 5 ng/mL) for 60 min, followed by 6 h of PDGF-BB treatment. Expression levels of TM and Ets-1 were analyzed with Western blot and normalized by GAPDH. Western blots from three independent experiments were analyzed by densitometry. The indicated fold changes represent the density relative to control (-),* p < 0.05 compared with 0 ng/mL PDGF-BB and rapamycin. (E) Immunofluorescence staining of TM in MCECs with or without 10 ng/mL PDGF-BB treatment for 6 h. Data represent means ± SD. TM (green), DAPI (blue). Magnification: 200×. Bar = 100 μm.

To investigate the mechanism of TM expression in the process of corneal epithelial wound healing, primary MCECs were isolated and cultured via previously reported methods [19, 21, 22], with some modifications. We confirmed that the MCECs (third passage) expressed TM and the corneal epithelium specific marker K12 (Fig. 4A and 4B). To explore whether PDGF-BB influences TM expression in MCECs, cells were starved for 24 h before stimulation with PDGF-BB for 6 h, and we also investigated the involvement of mTOR in TM induction by treating MCECs with rapamycin (0, or 5 ng/mL) for 60 min. Western blot analysis and immunofluorescence staining of TM showed that PDGF-BB induced TM expression in HCECs (Fig. 4C) and MCECs (Fig. 4D and 4E). Pretreatment with rapamycin resulted in the inhibition of PDGF-BB-induced TM expression (Fig. 4D). To evaluate the role of Ets-1 in PDGF-BB-induced TM expression, PDGF-BB-stimulated Ets-1 expression was examined with and without rapamycin (Fig. 4C and 4D). PDGF-BB treatment increased Ets-1 expression, which was inhibited by rapamycin.

### rTMD23 promoted corneal epithelial wound healing

**Fig 5:**
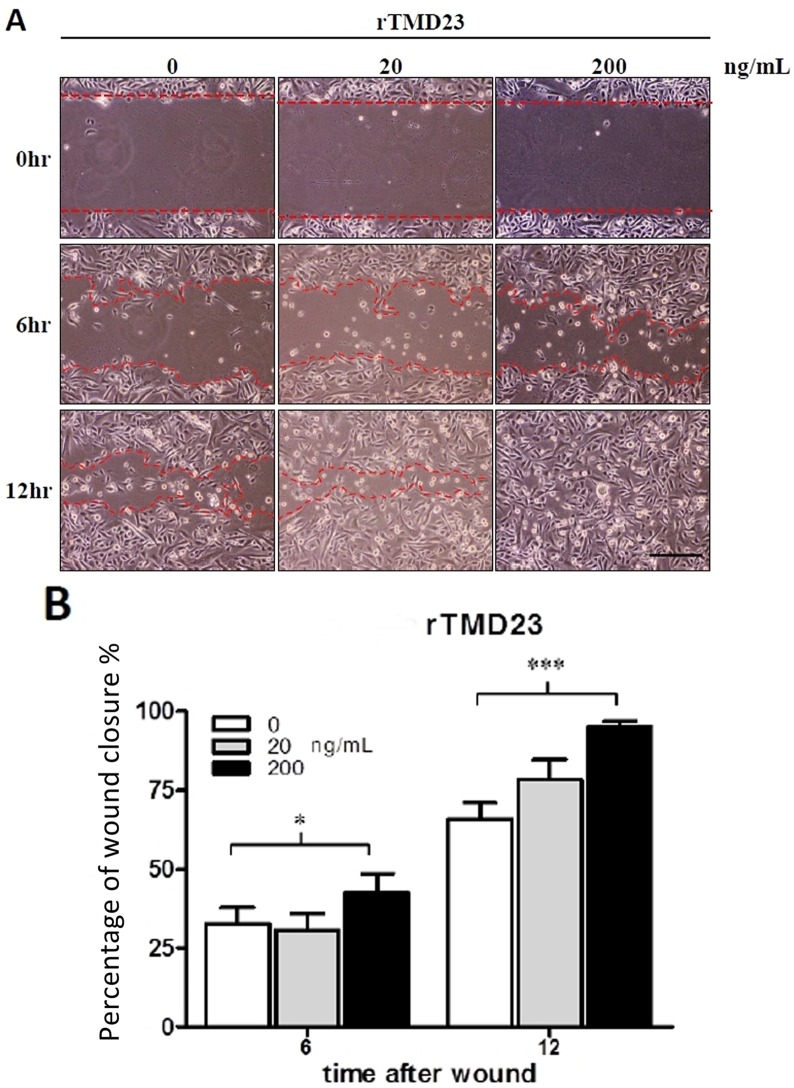
rTMD23 promotes wound closure in MCECs. *(A)* Representative phase microscopic images show wound closure at 0, 6, and 12 h. Growth factor-starved MCECs (third passage) were scraped and allowed to heal in the CnT-20 medium supplemented with different concentrations of rTMD23. *(B)* Statistical analysis of MCECs wound healing. The remaining acellular area of MCECs was photographed and measured at 0, 6, and 12 h after scraping. Data represent means ± SD. All experiments were performed in triplicates. * p < 0.05, *** p < 0.001 compared with 0 ng/mL rTMD23. Magnification: 100×. Bar = 250 μm.

**Fig 6:**
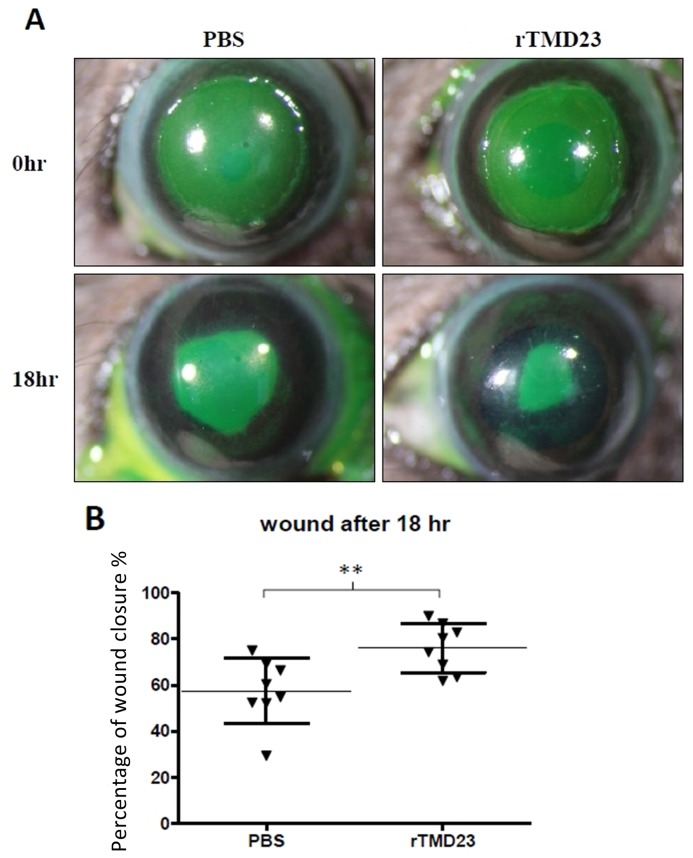
rTMD23 promotes wound healing in vivo. *(A)* A 2-mm wound was created on the cornea of an 8-week-old C57BL/6 mouse (n = 8/group). The mouse was given 5-μL (2 mg/mL) rTMD23 or 5-μL PBS at 3, 6, 9, 12, and 15 h after wounding. After 18 h, the wound area was stained by fluorescein. *(B)* Statistical analysis of the healing percentage of the wound area. Data represent means ± SD. The experiment was repeated three times. The result was analyzed using the unpaired *t*-test. ** p < 0.01 compared with PBS.

Previously, we demonstrated that rTMD23 promotes skin wound healing in mice [14]. In the current study, we investigated whether rTMD23 promoted corneal wound healing in vitro and in vivo. In the in vitro corneal epithelial wound healing assay, confluent MCECs were scratched and treated without or with rTMD23 (20 and 200 ng/mL), and the wound area was measured at 6 and 12 h post-wounding. All experiments were performed in triplicate. The percentages of wound closure exhibited by MCECs (third passage) cultured in medium with 200 ng/mL rTMD23 at 6 and 12 h after wounding were 42.7% and 95.2% respectively. In MCECs cultured without rTMD23, the percentages of wound closure after 6 and 12 h were 32.7% and 65.8% respectively. The percentages of wound closure were significantly greater in the rTMD23 treated group (*p* < 0.05) compared with the untreated controls (Fig. 5B, *n* = 3). For the in vivo corneal wound healing assay, a wound 2 mm in diameter was generated and the mouse was treated with 5 μL of 2 mg/ml rTMD23 at 3, 6, 9, 12, and 15 h after wounding. The wound area was measured by fluorescence staining 18 h after wounding (Fig. 6A). The mean percentage of wound closure in eyes treated with rTMD23 was 74.4%, which was significantly higher than the 54.9% observed in the PBS-treated group (*n* = 8/group, *p* < 0.01, Fig. 6B). Taken together, these data demonstrated that rTMD23 promoted corneal epithelial wound healing in MCECs and the murine model of corneal debridement wounding.

## Discussion

TM plays a critical role in the wound healing process [14]; however, the biological function of TM in corneal wound healing remains undetermined. In this study, we first established an animal model to examine TM distribution in the eyes of C57BL/6 mice. Our results indicate that TM distribution in the murine eye is similar to that in humans, in whom TM was reportedly detected in corneal epithelium, endothelium, limbus, iris, and ciliary body (Fig. 1) [15]. Furthermore, we found that TM expression was stronger in corneal epithelial basal cells than in epithelial wing and superficial cells (Fig. 2C). Corneal epithelial basal cells reportedly originate from limbal stem cells, then undergo proliferation, migration, and differentiation to maintain corneal epithelium homeostasis [23]. Our finding of TM being primarily expressed in corneal epithelial basal cells suggests that TM may be associated with corneal epithelium homeostasis.

We used a corneal debridement model to investigate the involvement of TM expression on corneal wound healing. We have previously reported that TM can promote and regulate skin wound healing, that TM knockout in the epidermis delays wound recovery in vivo, and that TM-knockout keratinocytes exhibit decreased spreading and slow migration ability [14]. In this current study, we observed that TM expression was increased in the early phase of the corneal epithelial wound healing process, and decreased after wound recovery (Fig. 3). In Fig. 3, images generated using the same fluorescence exposure time to determine TM expression in the corneal wound healing process are displayed. Compared with the increased TM expression in the whole corneal epithelium layer at 6 and 18 h post-wounding, TM staining was relatively weaker in the unwounded cornea and healed cornea. In addition, TM was expressed in the migrating and proliferating epithelium cells in whole epithelial layers during wound healing, indicating that TM may participate in the corneal epithelial wound healing process by regulating migration and proliferation.

To determine the role of TM in corneal epithelial cells, we isolated and cultured primary MCECs using a previously described method [19, 21, 22], and also used HCECs for experiments. We demonstrated that TM was expressed in MCECs (Fig. 4A and 4B) and found that PDGF-BB could stimulate TM expression in HCECs and MCECs (Fig. 4C and 4D). Previous studies have suggested that growth factors such as EGF, TGF-β, and PDGF released into tears may play an important role during corneal wound healing [24, 25]. Among these, PDGF receptor is expressed in the human corneal epithelium, inducing the migration and proliferation of corneal cells after wounding [26–29]. PDGF-BB can also stimulate TM expression in the corneal stromal and scleral stromal cells in inflammatory diseases such as endophthalmitis and herpetic keratitis [16]. In a previous study, we found that PDGF-BB stimulates functional TM expression by mediating Ets-1 via the mTOR signaling pathway [20]. In the current study, we identified the same mechanism in the corneal epithelial wound healing process. Our results show that the inhibition of mTOR repressed both PDGF-BB-induced Ets-1 upregulation and TM expression (Fig. 4D). We suggest that PDGF-BB induces TM expression by upregulating the transcription factor Ets-1 via the mTOR signaling pathway during corneal wound healing.

In the corneal wound healing assay, rTMD23 promoted corneal wound healing in vitro and in vivo (Fig. 5 and 6). Previously, rTMD23 was considered an angiogenic factor, enhancing the angiogenic response [17] and promoting mouse cutaneous wound healing through modulating angiogenesis at the wound site [13, 14]. However, we did not observe any neovascularization in the cornea after rTMD23 treatment in this current study. Our results indicate that rTMD23 promoted corneal wound healing, but not via angiogenic activity. Given that cell migration and proliferation play important roles during corneal epithelial wound healing [30, 31], rTMD23 has mitogenic activity for Swiss 3T3 cells [32], and rTMD23 enhances proliferation and migration in endothelial cells [17], we suggest that rTMD23 promotes corneal epithelial wound healing by accelerating epithelial cell migration and proliferation.

## Conclusion

This is the first report to examine the TM distribution pattern in the C57BL/6 mouse eye, and the first paper to investigate the role of TM in the wound healing process of the corneal epithelium. TM expression was increased during the early phase of corneal epithelial wound healing and decreased after wound recovery. In addition, we found that TM expression in the corneal epithelial cells was regulated by PDGF-BB and Ets-1 via the mTOR signaling pathway. Finally, rTMD23 promoted corneal epithelial wound healing in MCECs and in our corneal epithelial debridement mouse model, suggesting that rTMD23 has therapeutic potential in corneal injury.
